# Antibody and T Cell Responses to *Fusobacterium nucleatum* and *Treponema denticola* in Health and Chronic Periodontitis

**DOI:** 10.1371/journal.pone.0053703

**Published:** 2013-01-15

**Authors:** Jieun Shin, Sang-A Kho, Yun S. Choi, Yong C. Kim, In-Chul Rhyu, Youngnim Choi

**Affiliations:** 1 Department of Immunology and Molecular Microbiology, BK21 CLS, School of Dentistry and Dental Research Institute, Seoul National University, Seoul, Korea; 2 Department of Periodontology, BK21 CLS, School of Dentistry and Dental Research Institute, Seoul National University, Seoul, Korea; University of Cape Town, South Africa

## Abstract

The characteristics of the T cell response to the members of oral flora are poorly understood. We characterized the antibody and T cell responses to FadA and Td92, adhesins from *Fusobacterium nucleatum*, an oral commensal, and *Treponema denticola*, a periodontal pathogen, respectively. Peripheral blood and saliva were obtained from healthy individuals and patients with untreated chronic periodontitis (CP, n = 11 paris) and after successful treatment of the disease (n = 9). The levels of antigen-specific antibody were measured by ELISA. In plasma, IgG1 was the most abundant isotype of Ab for both Ags, followed by IgA and then IgG4. The levels of FadA-specific salivary IgA (sIgA) were higher than Td92-specific sIgA and the FadA-specific IgA levels observed in plasma. However, the periodontal health status of the individuals did not affect the levels of FadA- or Td92-specific antibody. Even healthy individuals contained FadA- and Td92-specific CD4^+^ T cells, as determined by the detection of intracytoplasmic CD154 after short-term *in vitro* stimulation of peripheral blood mononuclear cells (PBMCs) with the antigens. Patients with CP tended to possess increased numbers of FadA- and Td92-specific CD4^+^ T cells but reduced numbers of Td92-specific Foxp3^+^CD4^+^ Tregs than the healthy subjects. Both FadA and Td92 induced the production of IFNγ and IL-10 but inhibited the secretion of IL-4 by PBMCs. In conclusion, *F. nucleatum* induced Th3 (sIgA)- and Th1 (IFNγ and IgG1)-dominant immune responses, whereas *T. denticola* induced a Th1 (IFNγ and IgG1)-dominant response. This IFNγ-dominant cytokine response was impaired in CP patients, and the Td92-induced IFNγ levels were negatively associated with periodontal destruction in patients. These findings may provide new insights into the homeostatic interaction between the immune system and oral bacteria and the pathogenesis of periodontitis.

## Introduction

The surface of the human body is colonized by billions of commensal bacteria that live in harmony with their host. According to the current paradigm, the host immune system maintains tolerance to commensals but develops active immunity against harmful pathogens. Vaughan *et al*. reported that *Neisseria meningitidis*, an opportunistic pathogen colonizing the upper respiratory tract, induces mucosal T and B cell memory, but *N. lactamica*, a commensal bacterium, induces only a T cell-independent B cell response in humans [1]. Similarly in mice, a lack of classical immunological memory is observed in the intestinal IgA response against gut commensals [2]. However, the commensal *Lactobacillus*, which colonizes the nasal mucosa and crosses the nasal epithelial barrier, induces Th1, but not Treg or Th2, responses in mice [3].

The human oral cavity harbors a complex bacterial community composed of more than 700 species [4]. Most are commensals, and only a small number of species are known to be associated with oral diseases, such as dental caries and periodontitis. Periodontitis is a polymicrobial disease that arises from a microbial shift in the indigenous flora of the plaque biofilm rather than from the sudden introduction of a new pathogen [5]. To understand the immunopathogenesis of periodontitis, antibody (Ab) and T cell responses to periodontal pathogens have been widely studied. A number of studies have demonstrated the presence of IgG Abs against periodontal pathogens using whole bacteria or isolated proteins, such as fimbriae from *Porphyromonas gingivalis*, bacterial surface protein A from *Tannerella forsythia*, and leukotoxin from *Aggregatibacter actinomycetemcomitans* in human serum or gingival crevicular fluid [6]–[10]. Two decades ago, the presence of T cells specific to *P. gingivalis* in the peripheral blood of patients with periodontitis and of healthy subjects was shown by a limiting dilution assay without further characterization of the bacteria-specific T cells [11]. In the following decade, several groups established peripheral blood and gingival T cell clones specific to periodontal pathogens and characterized their cytokine production [12]–[14]. Kopitar *et al*. recently reported that the commensal oral bacteria *Streptococcus mitis* and *Propionibacterium acnes* prime human dendritic cells to induce *in vitro* differentiation of Th2 and Treg cells, respectively [15]. However, all previous studies have employed long-term *in vitro* stimulation with bacterial antigens (Ags) prepared from whole bacteria or outer membranes, which allows for the activation of naïve T cells by specific Ags or non-specific mitogens [16]. Thus, the nature of adaptive immunity to oral comensals and periodontal pathogens in humans are poorly understood.

The aims of the current study include i) to characterize the T-dependent Ab and T cell responses to *Fusobacterium nucleatum*, an oral commensal, and *Treponema denticola*, a periodontal pathogen, in healthy individuals, and ii) to determine difference in those Ab and T cell responses between healthy subjects and patients with chronic periodontitis (CP). Although the levels of *F. nucleatum* are significantly associated with increasing pocket depth [17], *F. nucleatum* is a prevalent member of plaque biofilm in healthy individuals and is considered a commensal or possibly beneficial bacterium due to its ability to induce antimicrobial peptides in gingival epithelial cells [18], [19]. *T. denticola* is one of the major periodontal pathogens involved in CP [20]. To exclude components that are mitogenic or reactive to natural Abs produced in a T-independent manner, a single surface protein was chosen as a representative T-dependent Ag for each bacterium. FadA is an adhesin of *F. nucleatum* involved in adhesion and cellular invasion [21]. Td92 is an adhesin of *T. denticola* and an inducer of proinflammatory cytokines [22]. The vaccine protein tetanus toxoid (TT) was used as a control Ag to provide a comparison to a classical systemic immune response. Through the examination of Ab, Ag-specific CD4^+^ T cells, and the cytokine response to these three Ags, here, we demonstrate that both *F. nucleatum* and *T. denticola* normally induce T cell memory in humans and that CP is associated with a reduced IFNγ/IL-4 cytokine ratio. These findings may provide new insights into the homeostasis of the immune system with oral bacteria and the pathogenesis of periodontitis.

## Materials and Methods

### Study Subjects and Periodontal Assessment

Individuals with CP (n = 11) were recruited from patients who visited the Department of Periodontology at the Seoul National University Dental Hospital. CP was diagnosed based on the classification system established at the International Workshop for a Classification of Periodontal Diseases and Conditions in 1999 [23]. Age- and gender-matched healthy control subjects (n = 11) were recruited through an advertisement. Control subjects were designated as healthy if they had both full-mouth probing pocket depth and full-mouth clinical attachment level not greater than 3 mm. All healthy subjects had no history of periodontal treatment or diagnosis with periodontitis. All subjects were in good general health and had no history of antimicrobial or periodontal treatment for 3 months before the start of the study. The study was performed according to the principles expressed in the Declaration of Helsinki, after receiving approval from the Institutional Review Board of the Seoul National University Dental Hospital. Written informed consent was obtained from all subjects. After collecting 20 ml peripheral blood and 5 ml saliva, the full-mouth pocket depth, clinical attachment level from the cementoenalmel junction, and bleeding on probing at six sites for each tooth were recorded by one calibrated examiner. All clinical parameters were measured with a manual probe (CP-12, Hu Friedy, Chicago, IL, USA). Four weeks after receiving quadrant scaling/curettage (four visits within four to six weeks), nine patients who were successfully treated and participated in a re-call check were re-sampled.

### Human Materials

Plasma samples were obtained by centrifugation of 1 ml blood. The supernatant and solid pellet of saliva were separated. Plasma and the saliva supernatant were stored at −20°C until use. The solid pellet of saliva was subjected to DNA extraction. The remaining blood was layered on Ficoll-Hypaque (Amersham Bioscience, Uppsala, Sweden) after diluting in PBS at 1∶1 and centrifuged at 400 g for 40 min. PBMCs in the buffy coat layer were separated and stored in a liquid nitrogen tank until use.

### Recombinant FadA and Td92 Proteins

*Escherichia coli* M15 transformed with the Td92-containing pQE30 vector was kindly provided by Dr. Bong Kyu Choi at School of Dentistry, Seoul National University and the construction of Td92- containing pQE30 is previously reported [22]. The entire coding region for the FadA gene was amplified from the gDNA of *F. nucleatum* ATCC 25586 by PCR using the following primers: 5′-GGAATTCCATATGAAAAAATTTTTATTATTAGCAG-3′ and 5′-CCGCTCGAGGTTACCAGCTCTTAAAGCTTG-3′. The forward and reverse primers contained Nde I and Xho I sites (underlined), respectively. The amplified gene segments were cloned into the Nde I-Xho I sites of an expression vector pET-30(a) (QIAGEN, Valencia, CA, USA) and the plasmid was used to transform *E. coli* BL21. Expression of the recombinant proteins in *E. coli* was induced by treatment with 1 mM isopropyl-β-D-1-thiogalactopyranoside for 4 h. The recombinant FadA and Td92 were purified from the bacterial lysate using nickel-nitrilotriacetic acid agarose (Ni-NTA agarose; QIAGEN). The identities of the purified recombinant FadA and Td92 proteins were confirmed by SDS-PAGE gel electrophoresis and coomassie blue staining (Figure S1). The proteins were then dialyzed three times against 200 volumes of PBS for 24 h.

### Measurement of Ag-specific IgA, IgG1, and IgG4 in the Plasma and Saliva

High binding 96-well EIA/RIA plates were coated with 200 ng/ml FadA, Td92 or TT (GreenCross, Yongin, Kyungki, Korea) at 4°C overnight. For the standard, two columns were coated with serially diluted (30 ng/ml to 0.04 ng/ml) human IgA, IgG1, or IgG4 mAb (Southern Biotech, Birmingham, AL, USA) instead of Ag. After blocking with 1% BSA in PBS at room temperature for 1 h 30 min, the plates were incubated with 100 µl plasma or saliva samples at room temperature for 1 h 30 min. For each sample, three serially diluted samples were prepared. All samples and standards were run in duplicate. After washing, the plates were incubated with 100 µl HRP-conjugated goat-anti-human-IgA, -IgG1, or -IgG4 polyclonal Ab (Sothern Biotech) in 1% BSA-PBS for 1 h. After washing, the bound detection Ab was developed with 100 µl 3, 3′, 5, 5′-tetramethylbenzidine substrate (Sigma, St. Louis, MO, USA) for 15 min. After stopping the reaction with 2 N H_2_SO_4_, the plates were immediately read at 450 nm. The levels of Ag-specific Abs were calculated using an equation generated from the standard curve.

### Stimulation of PBMCs with Ag

PBMCs were thawed and stabilized in culture medium (RPMI 1640 medium with 10% heat-inactivated FBS, 2 mM L-glutamine, and 100 U/ml penicillin/streptomycin) at 37°C for 12 h. For flow cytometry analysis, PBMCs (2×10^6^ cells/200 µl/well) were stimulated with 30 µg/ml Ag protein, 2 µg/ml anti-CD28 mAb (BD Bioscience, San Diego, CA, USA), and 2 µg/ml anti-CD49d mAb (BD Bioscience) in the presence of 20 µl of PE-conjugated anti-CD154 mAb (BD Bioscience) at 37°C for 16 h. Monensin (2 µM, BD Bioscience) was added to the culture medium for the last 4 h prior to harvesting cells. To analyze effector cytokines produced in response to Ags, PBMCs (1×10^6^ cells/200 µl/well) were stimulated with 30 µg/ml Ag protein, 2 µg/ml anti-CD28 mAb, and 2 µg/ml anti-CD49d mAb for 48 h. The concentration of the Ag and the stimulation time were determined by preliminary experiments to obtain the maximal difference in the number of CD154^+^CD4^+^ T cells and cytokine production compared to unstimulated cells. After thawing and stabilization, the PBMCs samples that did not have enough live cells for the experiment were excluded from the assay.

### Cell Staining and Flow Cytometry Analysis

After stimulation, Ag-specific cells were identified by cytoplasmic staining of CD154. The harvested cells were blocked with 0.1% human serum and stained with APC-conjugated anti-CD4 mAb (BD Bioscience). Cells were then stained with FITC-conjugated anti-Foxp3 mAb (BD Bioscience) after permeabilization with Fix/Perm (BD Bioscience). Cells were analyzed with a FACSCalibur flow cytometer (BD Bioscience) using the CellQuest software (BD Bioscience). Lymphocytes gated based on forward and side scatters were plotted in the density plots of CD4 vs. CD154, and the percentage of CD154^+^ CD4^+^ T cells was determined. Then, the CD154^+^CD4^+^ T cells were gated and plotted against Foxp3 and CD4 to obtain the percentage of Ag-specific Foxp3^+^CD4^+^ T cells.

### Measurement of Cytokines

The amount of IL-4, IL-10, IL-17, and IFNγ secreted into the culture medium was measured using ELISA kits (R&D Systems, Minneapolis, MN, USA) according to the manufacturer’s instructions.

### Quantification of F. nucleatum and T. denticola in Saliva

To estimate the level of *T. denticola* and *F. nucleatum* in the oral cavity, the relative proportion of each bacterium in saliva was determined by real-time PCR. Bacterial genomic DNA was extracted from the solid pellet of saliva using a G-spin™ bacterial genomic extraction kit (iNtRON, Kyung-gi, Korea). One microgram of gDNA was subjected to real time-PCR amplification using universal primers that targeted a highly conserved region of 16s rRNA, and bacteria-specific primers that targeted a specific area in the 16s rRNA gene of *F. nucleatum* or *T. denticola*. A 20 µl reaction mix containing 1 µg gDNA, SYBR Premix Ex *Taq*, ROX Reference Dye (Takara Bio, Otsu, Japan), and each primer (0.2 µM) was prepared and subjected to amplification using a fluorescence thermocycler (Applied Biosystems 7500 Real-time PCR, Foster City, CA, USA) under the following conditions: initial denaturation at 94°C for 1 min, followed by 40 cycles of denaturation at 95°C for 15 s, annealing at 60°C for 15 s, and elongation at 72°C for 33 s. The specificity of the PCR product was verified by melting curve analysis. Relative copy numbers of *F. nucleatum* and *T. denticola* rRNA genes compared to the products of the universal rRNA primers were calculated using 2^−ΔCt^. Real-Time PCR was performed in triplicate for each gDNA sample. The primer sequences used are as follows: 5′-ACTCCTACGGGAGGCAGC-3′ and 5′-CAATTCCTTTGAGTTTCACC-3′ for the universal primers; 5′-TGTAGTTCCGCTTACCTCTTCAG-3′ and 5′-AAGCGCGTCTAGGTGGTTATGT-3′ for *F. nucleatum*; and 5′-TAATACCGAATGTGCTCATTTACAT-3′ and 5′-TCAAAGAAGCATTCCCTCTTCTTCTTA-3′ for *T. denticola*.

### Statistics

The differences between the healthy subjects and CP patients were analyzed by Mann-Whitney U test. The differences in response to two different Ags or before and after treatment for the same subjects were analyzed by Wilcoxon signed-rank test. Pairwise correlation analysis was performed using Spearman’s rank correlation test. All statistics were performed using the SPSS Statistics19 software (IBM, Seoul, Korea). Data were considered statistically significant at a *p*-value of <0.05.

## Results

### Clinical Parameters of Subjects

Compared to healthy subjects, patients with untreated CP had significantly increased levels of clinical attachment level, a parameter of periodontal destruction, and increased levels of bleeding on probing, a parameter of inflammation. Successful treatment of the disease improved both clinical parameters to the levels that are comparable to those of healthy subjects (Table 1).

**Table 1:** Characteristics and clinical parameters of subjects.

	Healthy	Patients (Before)	*P* value†	Patients (After)	*P* value‡
Sex (female %)	36	36		33	
Smoker (%)	18	18		22	
Age	34.1±11.8§	34.5±11.7	*P* = 0.867	32.3±3.2	*P* = 0.301
Clinical attachment level (mm)	2.0±0.4‡	3.4±0.2	*P*<0.0001	2.3±0.2	*P* = 0.078
Bleeding on probing (%)	5.2±4.4‡	66.6±32.1	*P*<0.0001	11.6±9.0	*P* = 0.122

† Mann-Whitney U test between healthy and patients with CP before treatment.

‡ Mann-Whitney U test between healthy and patients with CP after treatment.

§ Mean ± standard deviation.

### Ab Responses in Plasma and Saliva

B cells specific to T-dependent Ags undergo class switching with the help of follicular B helper T cells that provide CD40L and multiple cytokines. The production of IgG1, IgG4, and IgA involves IFNγ, IL-4, and TGFβ, respectively [24]–[26]. Because the cytokine secretion patterns of follicular B helper T cells and peripheral effector helper T cells are similar, the production of IgG1, IgG4, and IgA reflect Th1, Th2, and Th3 responses, respectively [27]. Therefore, we determined the levels of IgG1, IgG4, and IgA in the plasma and the levels of IgA in the saliva by ELISA to characterize both the Ab and T cell responses to the Ags.

We first analyzed the Ab responses mounted against the three Ags in healthy subjects. In the plasma, IgG1 was the most abundant isotype of Ab for all three Ags, followed by IgA and then IgG4. However, lower levels of FadA- and Td92-specific IgG1 and IgG4 were present compared to those specific to TT. The levels of Td92-specific IgG1 were particularly low. The levels of FadA-specific salivary IgA (sIgA) were significantly higher than the levels of either Td92- or TT-specific sIgA (Figure S2). For FadA-specific IgA, the levels in saliva were higher than those observed in plasma (*P* = 0.03).

**Figure 1:**
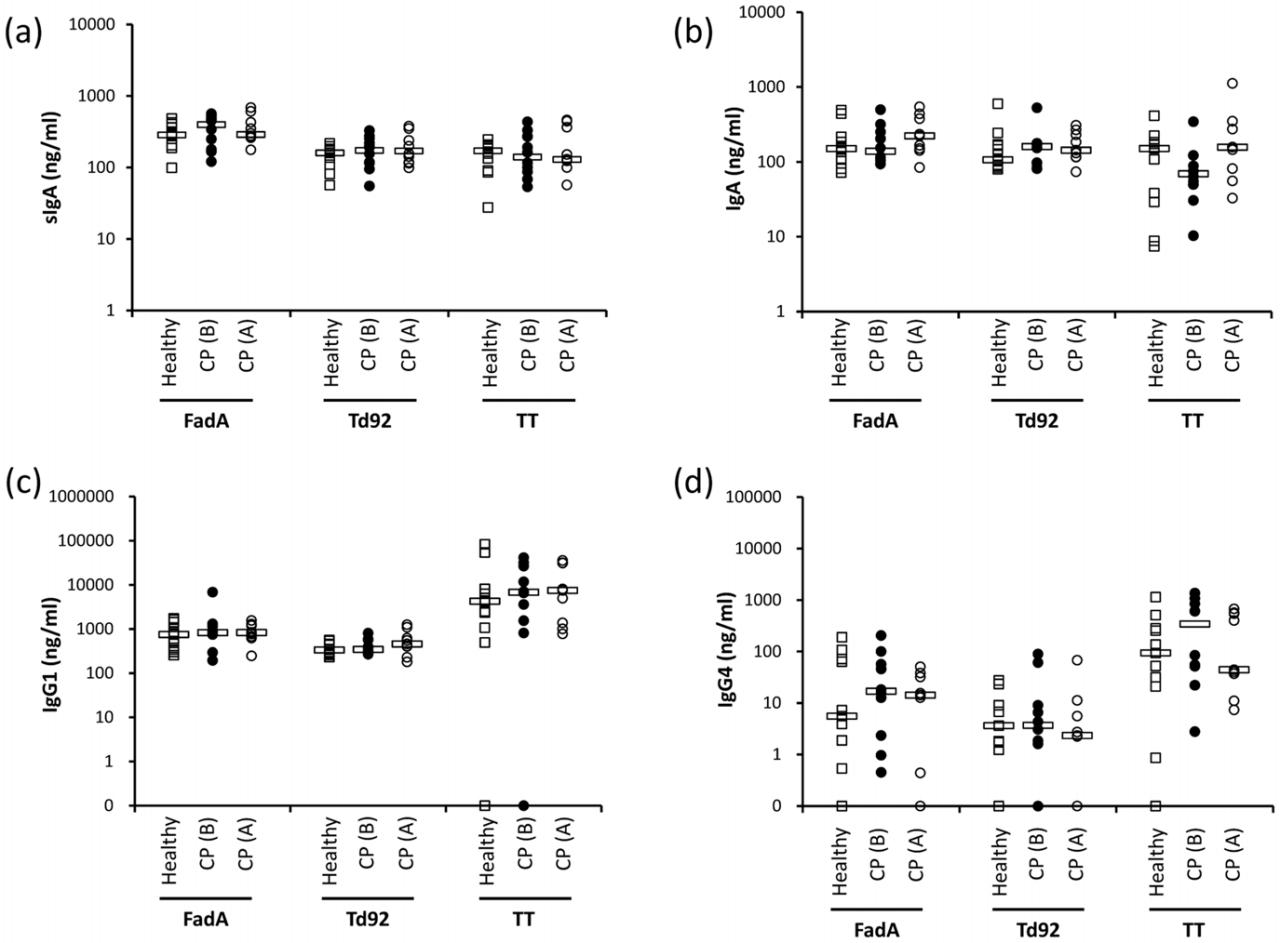
FadA-, Td92-, and TT-specific Abs in saliva and plasma. The levels of FadA-, Td92-, and TT-specific-IgA in saliva (a) and the Ag-specific-IgA (b), -IgG1 (c), and -IgG4 (d) in plasma were determined by ELISA. Bars indicate median values. CP (B): patients with chronic periodontitis before treatment. CP (A): patients with chronic periodontitis after treatment.

Next, the Ab responses of healthy subjects and CP patients were compared. Although slight increase in the levels of FadA- or Td92-specific sIgA, IgA, or IgG4 were observed in CP patients, the differences were not statistically significant. Similarly, the disease treatment did not significantly alter the levels of Abs compared with those before treatment of CP (Figure 1a, b, c, d).

These data show that different Ab responses were mounted against FadA, Td92, and TT, but the periodontal health status of the individuals had no significant effect on the levels of Abs against the three Ags.

### The Presence of Ag-specific CD4^+^ T cells and Foxp3^+^CD4^+^ Tregs

**Figure 2:**
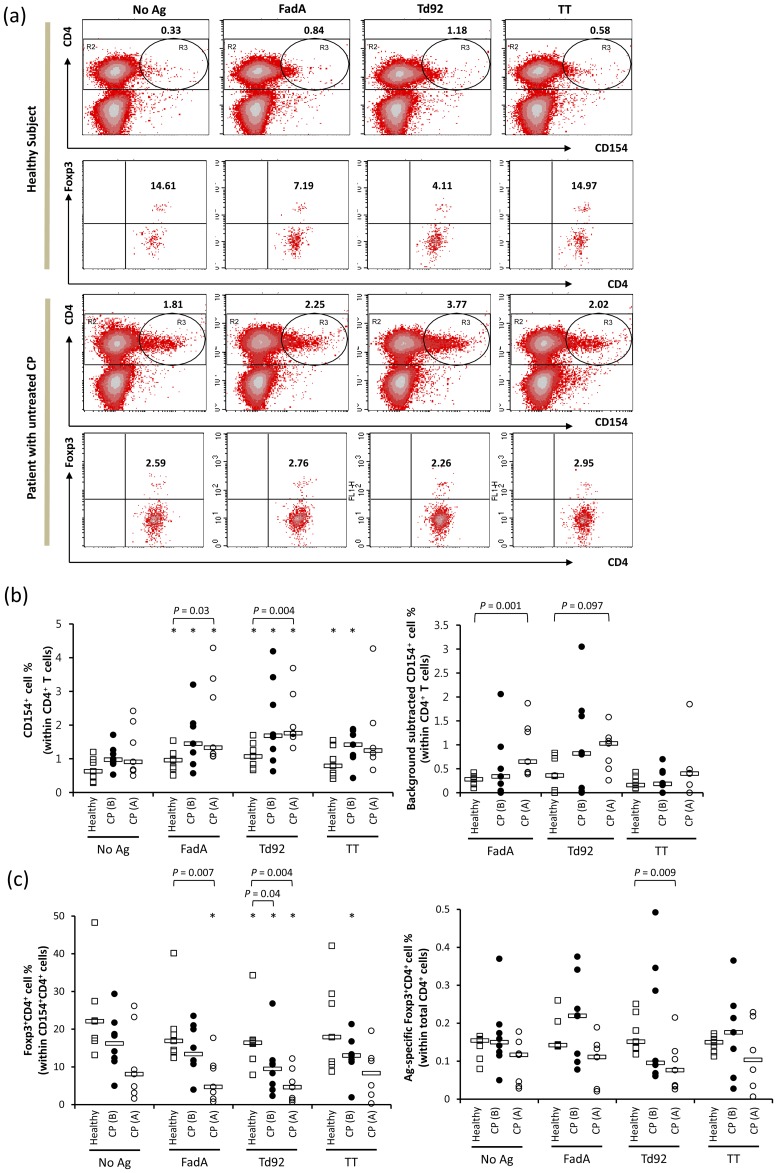
Presence of FadA-, Td92-, and TT-specific CD4^+^ T cells and Ag-specific Foxp3^+^CD4^+^ Tregs in PBMCs PBMCs from healthy subjects (n = 7), patients with CP before (B) treatment (n = 8), and patients with CP after (A) treatment (n = 7) were stimulated with medium alone (No Ag), 30 µg/ml FadA, Td92, or TT for 16 hours in the presence of PE-conjugated anti-CD154 mAb, purified anti-CD28 mAb, and anti-CD49d mAb. Monensin was added during the last 4 h of culture. The stimulated cells were stained with an APC-conjugated anti-CD4 mAb and a FITC-conjugated anti-Foxp3 mAb. (a) Lymphocytes gated based on FSC vs. SSC were plotted by CD4 vs. CD154 in the density plots (Top and the third rows). The percentage of CD154^+^ cells (R3) among the CD4^+^ T cells (R2) is presented. CD154^+^CD4^+^ T cells were gated and plotted by Foxp3 vs. CD4 (the second and the fourth rows). Numbers indicate the percentage of Foxp3^+^ cells among the gated CD154^+^CD4^+^ T cells. (b) The percentage of CD154^+^ cells out of the total CD4^+^ T cells was graphed (left panel). The percentage of Ag-specific cells out of the total CD4^+^ T cells was graphed after subtracting the number of background stained cells (right panel). (c) The percentage of Foxp3^+^CD4^+^ Tregs among the CD154^+^CD4^+^ T cells was graphed (left panel). The percentage of CD154^+^Foxp3^+^CD4^+^ T cells out of the total CD4^+^ T cells was graphed (right panel). Bars indicate median values. *, *P*<0.05 compared to No Ag.

The presence of Ag-specific CD4^+^ T cells was determined by the detection of intracytoplasmic CD154 (CD40L) following short-term *in vitro* stimulation of peripheral blood mononuclear cells (PBMCs) with Ags [28], [29]. CD154 is transiently expressed on recently activated CD4^+^ T cells and is regarded to be a reliable intracytoplasmic marker of Ag-specific CD4^+^ T cells [30], [31]. Because regulatory T (Treg) cells also upregulate CD154 upon activation [32], the proportion of Tregs among the Ag-specific CD4^+^ T cells was simultaneously determined by intracellular Foxp3 staining (Figure 2a).

A small number of unstimulated CD4^+^ T cells were positive for CD154. These cells likely represent circulating effector cells that were activated *in vivo*. Stimulation with FadA, Td92, or TT significantly increased the number of CD154^+^CD4^+^ T cells in both healthy and CP groups (Figure 2b left panel), which suggests the presence of memory CD4^+^ T cells specific to these Ags. After subtracting the number of background stained cells, the mean percentages of FadA-, Td92-, and TT-specific cells out of the total CD4^+^ T cells in healthy subjects were 0.26±0.04, 0.42±0.14, and 0.21±0.05, respectively (Figure 2b right panel). There was no significant difference in the number of Ag-specific CD4^+^ T cells among the three Ags. Compared with healthy subjects, CP patients tended to contain increased numbers of CD154^+^CD4^+^ T cells for all three Ags and also for background (Figure 2a and b left panel). After subtracting the number of background stained cells, the mean percentages of FadA-, Td92-, and TT-specific cells out of the total CD4^+^ T cells in patients with untreated CP were 0.55±0.24, 1.02±0.38, and 0.14±0.17, respectively (Figure 2b right panel). CP treatment further increased the numbers of FadA- and Td92-specific CD4^+^ T cells, resulting in significant differences from the levels observed in healthy subjects (Figure 2b).

In healthy subjects, the percentage of Foxp3^+^CD4^+^ Tregs out of the Ag-specific CD154^+^CD4^+^ T cells was decreased upon Ag stimulation (Figure 2c left panel). No significant differences were observed in the responses elicited by the various Ags. Interestingly, patients with CP presented with a reduced percentage of Foxp3^+^CD4^+^ Tregs out of the Ag-specific cells in both the unstimulated and Ag-stimulated conditions. This difference was increased upon clinical therapy, which resulted in a significant difference from healthy subjects upon stimulation with FadA or Td92 (Figure 2c left panel). Because the number of Ag-specific CD4^+^ T cells was increased in CP patients, the percentage of Ag-specific Foxp3^+^CD4^+^ Tregs was adjusted to the total CD4^+^ T cell population. The expansion of Ag-specific Foxp3^+^CD4^+^ Tregs was examined in several patients with untreated CP. However, after therapy, the patients presented lower levels of Td92-specific Foxp3^+^CD4^+^ Tregs than the healthy subjects (Figure 2c right panel).

In summary, the treated CP patients presented increased levels of FadA- and Td92-specific CD4^+^ T cells but reduced levels of Td92-specific Tregs than the healthy subjects.

### Cytokine Response

We examined Ag-induced production of IFNγ, IL-4, and IL-17 that are Th1, Th2, and Th17 effector cytokines, respectively, in addition to a regulatory cytokine IL-10. Although identification of Ag-specific CD4^+^ T cells via flow cytometric detection of CD154 allows for the concurrent examination of cytokines, such as IL-2, TNFα, and IFNγ, the expression of IL-4 and IL-10 requires sorting and additional stimulation for 36 h [29], as confirmed in our preliminary study. Therefore, PBMCs were stimulated with Ags for 48 h, a time period that allows activation of memory T cells but limits that of naïve T cells, and the amounts of IFNγ, IL-4, IL-10, and IL-17 secreted into the supernatant were measured by ELISA. In healthy subjects, all three Ags increased the production of IFNγ and IL-10, and FadA and Td92 reduced IL-4 secretion compared to the basal levels (Figure S2). No significant change in the levels of IL-17 was observed upon Ag stimulation. TT induced the highest levels of IFNγ production among the three Ags, followed by Td92 and FadA. The levels of FadA-stimulated IL-17 production were lower than those induced by Td92 or TT (Figure S3).

**Figure 3:**
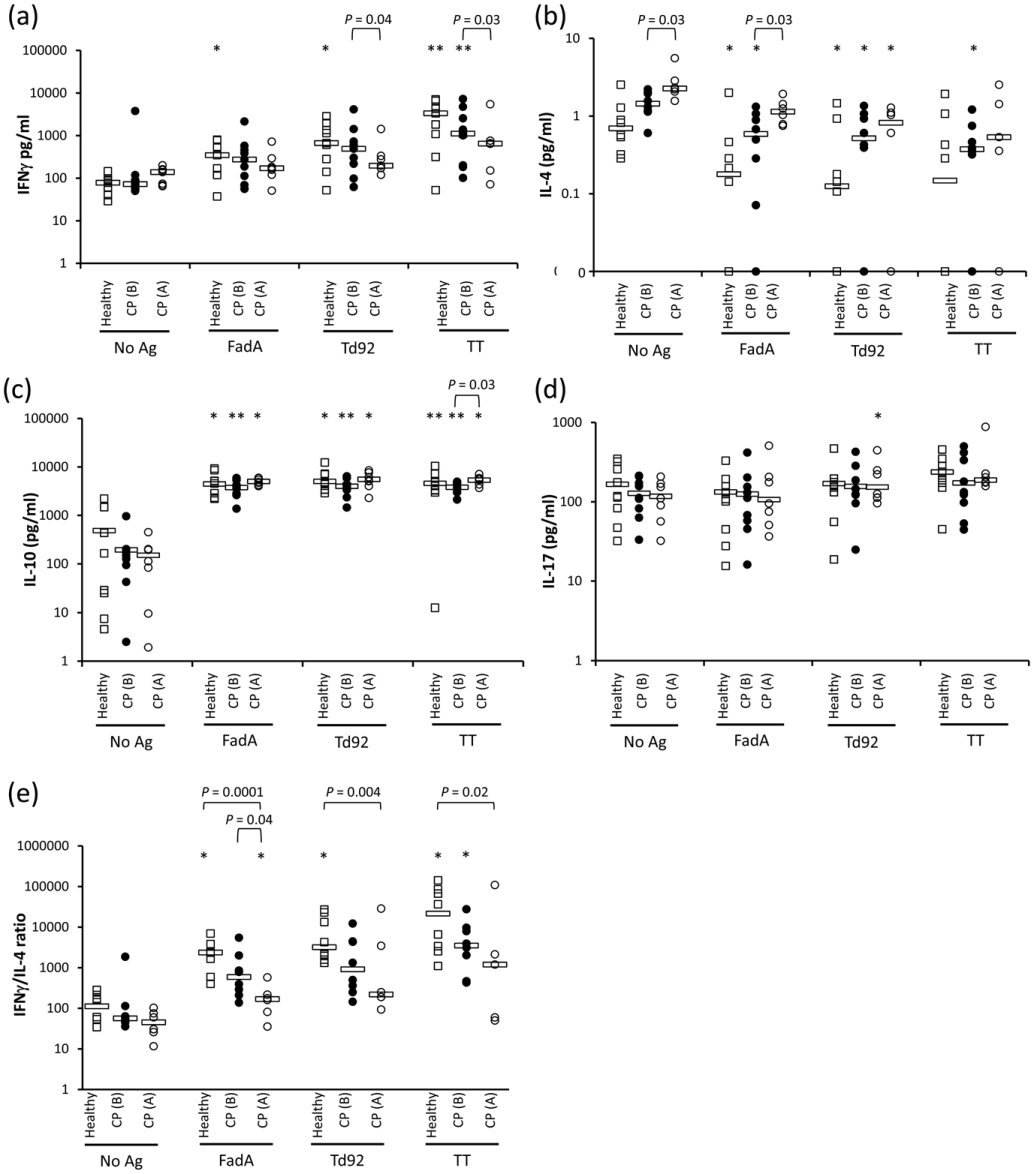
Cytokine production by PBMCs in response to FadA, Td92, or TT PBMCs from healthy subjects (n = 9), patients with CP before (B) treatment (n = 9), and patients with CP after (A) treatment (n = 7) were stimulated with medium alone (No Ag), 30 µg/ml FadA, Td92, or TT for 48 hours in the presence of purified anti-CD28 mAb and anti-CD49d mAb. (a–d) The amount of IFNγ, IL-4, IL-10, and IL-17 secreted into the supernatant was measured by ELISA. (e) The ratio of IFNγ/IL-4 was calculated and plotted. For the calculation of this ratio, half of the lowest detectable concentration (0.1 pg/ml) was arbitrarily assigned to IL-4 samples that had a zero value. Bars indicate median values. *, *P*<0.05; **, *P*<0.01 compared to No Ag.

Compared to healthy controls, PBMCs from CP patients failed to show a significant IFNγ response to either FadA or Td92 and tended to produce increased levels of IL-4 both at basal levels and following Ag stimulation. In addition, the patient PBMCs presented a tendency to produce consistently lower levels of IL-10 (Figure 3a, b, c, d). After clinical therapy, the differences in IFNγ and IL-4 levels were exaggerated, but the levels of IL-10 were restored (Figure 3a, b, c, d). Consequently, the patients with treated CP exhibited significantly reduced IFNγ/IL-4 ratios in response to all three Ags compared to the healthy subjects (Figure 3e).

These results indicate that different cytokine responses were induced by different Ags and by differences in the periodontal health status.

### Relative Amounts of F. nucleatum and T. denticola in Saliva

**Figure 4:**
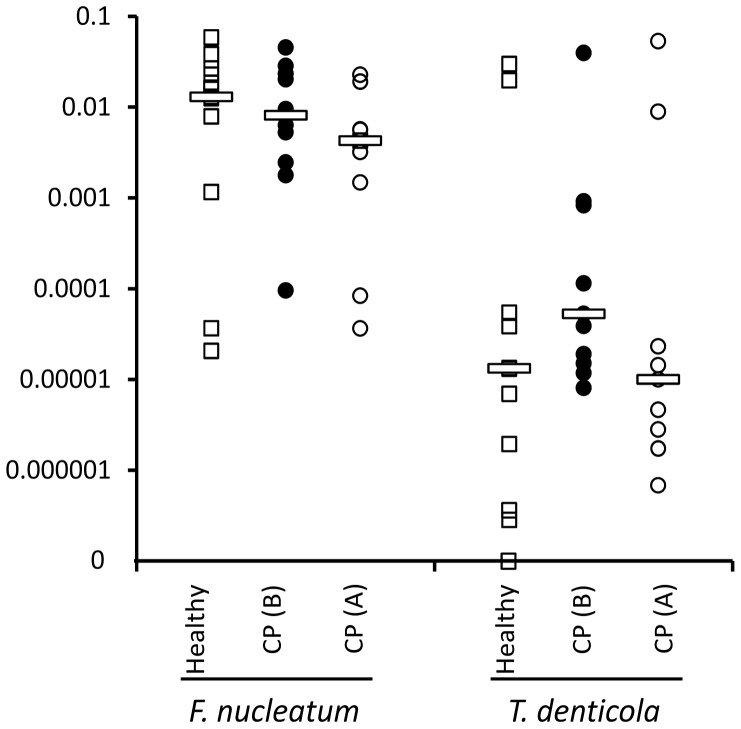
Relative amounts of F. nucleatum and T. denticola in saliva. The relative proportion of *F. nucleatum* or *T. denticola* among the total bacteria in saliva was determined by real-time PCR of 16S rRNA. Bars indicate median values.

To estimate the amounts of *F. nucleatum* and *T. denticola* colonizing the oral cavity, the relative proportion of each bacterium among total bacteria in saliva was determined by real-time PCR of 16S rRNA genes. With the exception of one individual, all healthy subjects carried detectable levels of *T. denticola*. Both healthy individuals and CP patients carried higher amounts of *F. nucleatum* than *T. denticola* (*P*<0.0001). Although statistical significance was not achieved, patients with CP contained a higher proportion of *T. denticola* than healthy subjects, which was restored by clinical therapy (Figure 4).

### Correlations among Clinical, Immunological, and Microbial Parameters

Correlations among the examined periodontal, immunological, and microbial parameters were analyzed. We first asked if there were any parameters associated with periodontal destruction. The relative proportion of *T. denticola* had a positive correlation with CAL (*P* = 0.03) in the combined population of healthy subjects and patients, but not in healthy group alone. In patients with untreated CP, the levels of Td92-induced IFNγ production and Td92-specific IgA in plasma had a negative and a positive correlation with CAL, respectively (*P* = 0.019 and *P* = 0.02, respectively). Meanwhile, the levels of Td92-specific sIgA were negatively associated with CAL in healthy subjects (*P* = 0.039).

The increased proportion of Ag-specific CD4^+^ T cells and the altered cytokine profile observed in CP patients may be a result of increased levels of bacteria. However, this correlation was not detected (data not shown). Interestingly, for almost all examined immunologic parameters, there were strong correlations between the Ags for each parameter. For example, high levels of FadA-specific sIgA correlated with high levels of Td92- and TT-specific sIgA (Table 2). This finding suggests that a certain intrinsic feature of each individual affects the individual’s immune response to all Ags.

**Table 2:** Correlations of the examined immunologic parameters between the Ags.

	Td92-specific/induced	TT-specific/induced
FadA-specific IgA in saliva	0.908† (*P*<0.0001)	0.845 (*P*<0.0001)
Td92-specific IgA in saliva	–	0.914 (*P*<0.0001)
FadA-specific IgA in plasma	0.858 (*P*<0.0001)	0.464 (*P* = 0.01)
Td92-specific IgA in plasma	–	0.453 (*P* = 0.012)
FadA-specific IgG1 in plasma	0.438 (*P* = 0.015)	0.069 (*P* = 0.716)
Td92-specific IgG1 in plasma	–	0.117 (*P* = 0.539)
FadA-specific IgG4 in plasma	0.249 (*P* = 0.184)	0.233 (*P* = 0.215)
Td92-specific IgG4 in plasma	–	0.433 (*P* = 0.017)
FadA-specific CD154^+^/CD4^+^ (%)	0.741 (*P*<0.0001)	0.944 (*P*<0.0001)
Td92-specific CD154^+^/CD4^+^ (%)	–	0.784 (*P*<0.0001)
FadA-specific Foxp3^+^/CD154^+^CD4^+^ (%)	0.816 (*P*<0.0001)	0.833 (*P*<0.0001)
Td92-specific Foxp3^+^/CD154CD4^+^ (%)	–	0.818 (*P*<0.0001)
FadA-specific Foxp3^+^/CD4^+^ (%)	0.808 (*P*<0.0001)	0.814 (*P*<0.0001)
Td92-specific Foxp3^+^/CD4^+^ (%)	–	0.712 (*P*<0.0001)
FadA-induced IFNγ	0.929 (*P*<0.0001)	0.824 (*P*<0.0001)
Td92-induced IFNγ	–	0.870 (*P*<0.0001)
FadA-induced IL-4	0.691 (*P*<0.0001)	0.429 (*P* = 0.032)
Td92-induced IL-4	–	0.427 (*P* = 0.033)
FadA-induced IL-10	0.761 (*P*<0.0001)	0.908 (*P*<0.0001)
Td92-induced IL-10	–	0.754 (*P*<0.0001)
FadA-induced IL-17	0.859 (*P*<0.0001)	0.609 (*P* = 0.002)
Td92-induced IL-17	–	0.778 (*P*<0.0001)

† Spearman’s rho calculated using all values from healthy individuals and patients with CP before and after therapy.

## Discussion

Our results have revealed that humans normally develop T cell memory to both *F. nucleatum* and *T. denticola*, regardless of the periodontal health status, as evidenced by the presence of FadA- and Td92-specific CD4^+^ T cells, IgG, and IgA as well as Ag-induced IFNγ production by PBMCs. The presence of FadA- and Td92-specific CD4^+^ T cells was determined by detecting intracytoplasmic CD154. The addition of anti-CD154 Ab to PBMC cultures during short-term *in vitro* stimulation with Ag stains only the specific T cells that have been previously activated with the Ag [30]. The CD154-stained Ag-specific T cells include CCR7^+^CD27^+^ central memory (T_CM_) and CCR7^-^CD27^+/−^ effector memory T (T_EM_) cells, and both populations contribute to cytokine production [30], [31]. The presence of FadA- and Td92-specific CD4^+^ T cells in PBMCs indicates that *F. nucleatum* and *T. denticola* can reach the systemic immune system to activate T cells, which may be attributable to the ability of these bacteria to invade gingival epithelial cells and/or gingival tissue [33], [34]. Alternatively, transient bacteremia, which can be triggered by mastication, tooth brushing, and various dental procedures, might be the route by which oral bacteria encounter the systemic immune system [35]. The increased number of FadA- and Td92-specific CD4^+^ T cells in patients, particularly after CP treatment, may reflect frequent bacteremia and the repeated activation of specific T cells. Compared to TT, FadA and Td92 induced lower levels of plasma IgG1 and IgG4 *in vivo*, and this coincides with the fact that both FadA and Td92 induced low levels of IFNγ production and down-regulated IL-4 production by PBMCs *in vitro*. When the FadA- and the Td92-induced responses were compared, significantly higher levels of FadA-specific sIgA and plasma IgG1 were observed, while Td92 induced higher levels of IFNγ and IL-17 production by PBMCs. Therefore, the immune evasive property of *T. denticola* reported in the innate immune compartment [19], [36] was not observed in the adaptive immune compartment. These results also indicate that *T. denticola* preferentially induces a cell-mediated rather than a humoral response. *F. nucleatum* seems to be efficient in inducing mucosal immunity as evidenced by the high levels of FadA-specific sIgA. Collectively, *F. nucleatum* induces Th3 (sIgA)- and Th1 (IFNγ and IgG1)-dominant immune responses, whereas *T. denticola* induces a Th1 (IFNγ and IgG1)-dominant immune response. One limitation of this study is that the presence of Ag-specific Th1 or Th3 cells was not directly shown. Because PBMC were used to examine cytokine profiles of Th subsets, CD8^+^ T cells and NK cells could have contributed to the IFNγ production.

The Th1-response to FadA and Td92 observed in healthy individuals raises the question how our immune system maintains homeostasis with the *F. nucleatum* and *T. denticola* present in the oral cavity. The epithelial barrier and sIgA would normally prevent bacterial invasion and the constant activation of T cells by these bacteria. The negative correlation between Td92-specific sIgA and CAL observed in healthy subjects suggests a protective role of sIgA in disease onset. If the oral bacteria invade epithelia and underlying tissue, another potential mechanism to maintain homeostasis would be regulation via regulatory cells and cytokines. The numbers of FadA- and Td92-specific Tregs within the total CD4^+^ T cells were comparable to the numbers of TT-specific Tregs. However, no significant difference observed in this study may be attributable to small sample size. Dendritic cells (DCs) conditioned by mucosal epithelial cells support the induction of several types of Tregs, including CD4^+^CD25^+^Foxp3^+^ induced Treg (iTreg), CD4^+^CD25^-^Foxp3^-^LAP^+^ Th3, and CD4^+^CD25^-^Foxp3^-^IL-10^+^ Tr1 [37]. Thus, there is a possibility that the FadA- or Td92-specific CD4^+^ T_CM_ cells may preferentially differentiate in the draining lymph nodes of the oral cavity *in vivo* into various types of Tregs. The low levels of FadA- and Td92-specific IgG1, IgG4, and IFNγ responses than TT-specific responses support this possibility. Ag stimulation of PBMCs induced high levels of the regulatory cytokine IL-10, but TT also elicited comparable levels of IL-10. Therefore, there is no sufficient evidence for or against the preferential regulation of immune response to FadA or Td92 through Tregs or IL-10. The best strategy to maintain the homeostatic relationship between the immune system and oral bacteria may be to keep bacteria outside tissue by competent epithelial barriers and sIgA.

Another important finding is the association of CP with a IFNγ/IL-4 cytokine imbalance. A number of conflicting results, including the dominance of Th1 cytokines [38], [39], of Th2 cytokines [40], [41], or no dominance [42], [43] in the periodontal lesions, have been reported over the last 15 years. These reports have recently been reviewed, and it was suggested that a skewed balance toward Th2 is responsible for periodontitis expression [44]. Our study coincides with the Th2-skewed response to oral bacterial Ags. Considering that periodontitis is a polymicrobial disease, the responses to two recombinant proteins from two oral species may not reflect the whole picture of immune response associated with periodontitis. In our study, however, the IFNγ/IL4 imbalance was also observed in response to TT, an Ag irrelevant to periodontitis. Furthermore, the levels of FadA-induced IFNγ or IL-4 had strong correlations with those induced by Td92 and TT in the combined population of healthy subjects and patients. These findings suggest that the skewed response toward Th2-like type observed in the CP patients may be an intrinsic feature of each subject rather than an Ag-specific phenotype. Increased IL-5 production by PBMCs from patients with CP following stimulation with mitogen also supports the intrinsic nature of the Th2 bias in CP patients [45]. Importantly, the levels of Td92-induced IFNγ had a negative correlation with CAL in patients, suggesting a protective role of the Th1 response to *T. denticola* in disease progression. Similarly, the importance of the Th1-dependent immune clearance of *P. gingivalis* for protection against alveolar bone loss has been demonstrated in a mouse model of periodontitis [46]. The levels of IL-4 production did not correlate with any clinical parameters. Whether the IFNγ/IL-4 imbalance is the cause or the result of disease warrants further investigation in larger samples.

Another important question to be answered in the future is to what degree the T cell responses observed in PBMCs reflect those in gingival tissues. The T_EM_ cells from PBMCs maintain effector functions of heterogeneous Th subsets, such as Th1, Th2, and Th17 [47], [48]. T_CM_ cells include committed pre-Th 1 and pre-Th 2 in addition to non-polarized cells [49]. Because the distinct cytokine profiles of Th or pre-Th subsets are maintained upon restimulation in the absence of polarizing factors [49], the IFNγ/IL-4 imbalance observed in the current study is likely to reflect that of the circulating memory T cell pool in each subject. Although the presence of Th17 cells in periodontal lesions has been reported [50], no IL-17 response to either FadA or Td92 was evident in our study using PBMCs. A study using gingival and peripheral blood T cells in parallel would clarify the relationship of Ag-specific T cells distributed in two compartments.

In conclusion, both *F. nucleatum* and *T. denticola* activate CD4^+^ T cells and induce a Th1-type response in humans. CP is associated with an impaired IFNγ/IL-4cytokine balance. These findings may provide new insights into the homeostatic interaction between the immune system and oral bacteria and the pathogenesis of periodontitis.

## Supporting Information

Figure S1 **Recombinant Td92 and FadA proteins.** The identities of the purified recombinant FadA and Td92 proteins were confirmed by SDS-PAGE gel electrophoresis and coomassie blue staining.
(PPTX)

Figure S2 **Comparison of Ab response to FadA, Td92, and TT in healthy individuals.** From the results shown in Figure 1, the data of healthy subjects are graphed separately. *, P<0.05; **, P<0.01.
(PPTX)

Figure S3 **Comparison of cytokine response of PBMCs to FadA, Td92, and TT in healthy individuals**. From the results shown in Figure 3, the data of healthy subjects are graphed separately. *, P<0.05; **, P<0.01 compared to No Ag. #, P<0.05; ##, P<0.01.
(PPTX)
