# Supplementary figures and images for: Antibody and T Cell Responses to Fusobacterium nucleatum and Treponema denticola in Health and Chronic Periodontitis
Source: PLoS One. 2013 Jan 15;8(1):e53703. doi: 10.1371/journal.pone.0053703 (PMC3546045; doi:10.1371/journal.pone.0053703)

## Slide 1
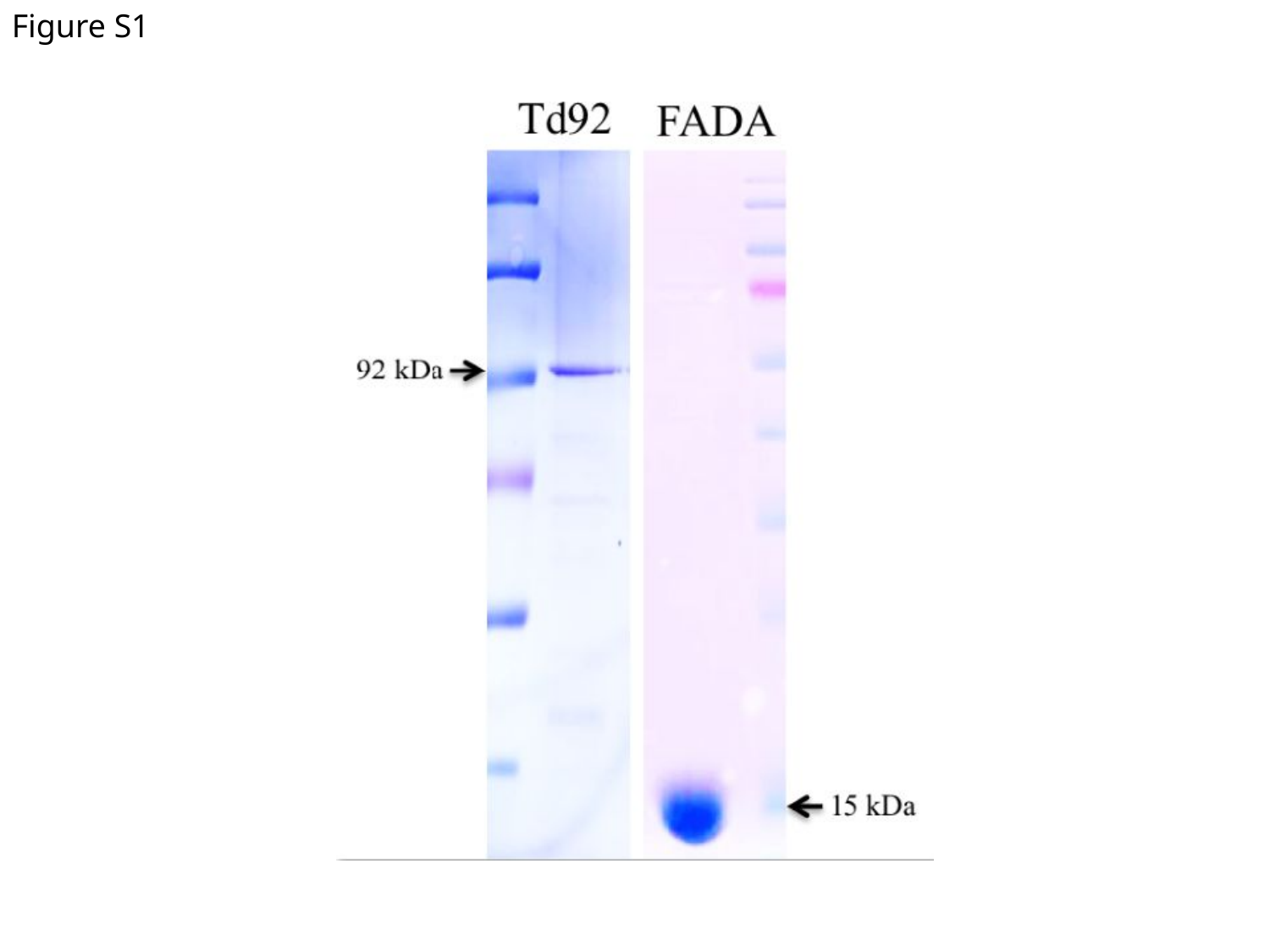

Figure S1

Supplement: Figure S1 — Recombinant Td92 and FadA proteins. The identities of the purified recombinant FadA and Td92 proteins were confirmed by SDS-PAGE gel electrophoresis and coomassie blue staining. (PPTX) [file pone.0053703.s001.pptx]
