# Supplementary material for: Antibody and T Cell Responses to Fusobacterium nucleatum and Treponema denticola in Health and Chronic Periodontitis
Source: PLoS One. 2013 Jan 15;8(1):e53703. doi: 10.1371/journal.pone.0053703 (PMC3546045; doi:10.1371/journal.pone.0053703)
Supplement: Figure S2 — Comparison of Ab response to FadA, Td92, and TT in healthy individuals. From the results shown in Figure 1, the data of healthy subjects are graphed separately. *, P<0.05; **, P<0.01. (PPTX) [file pone.0053703.s002.pptx]

## Slide 1
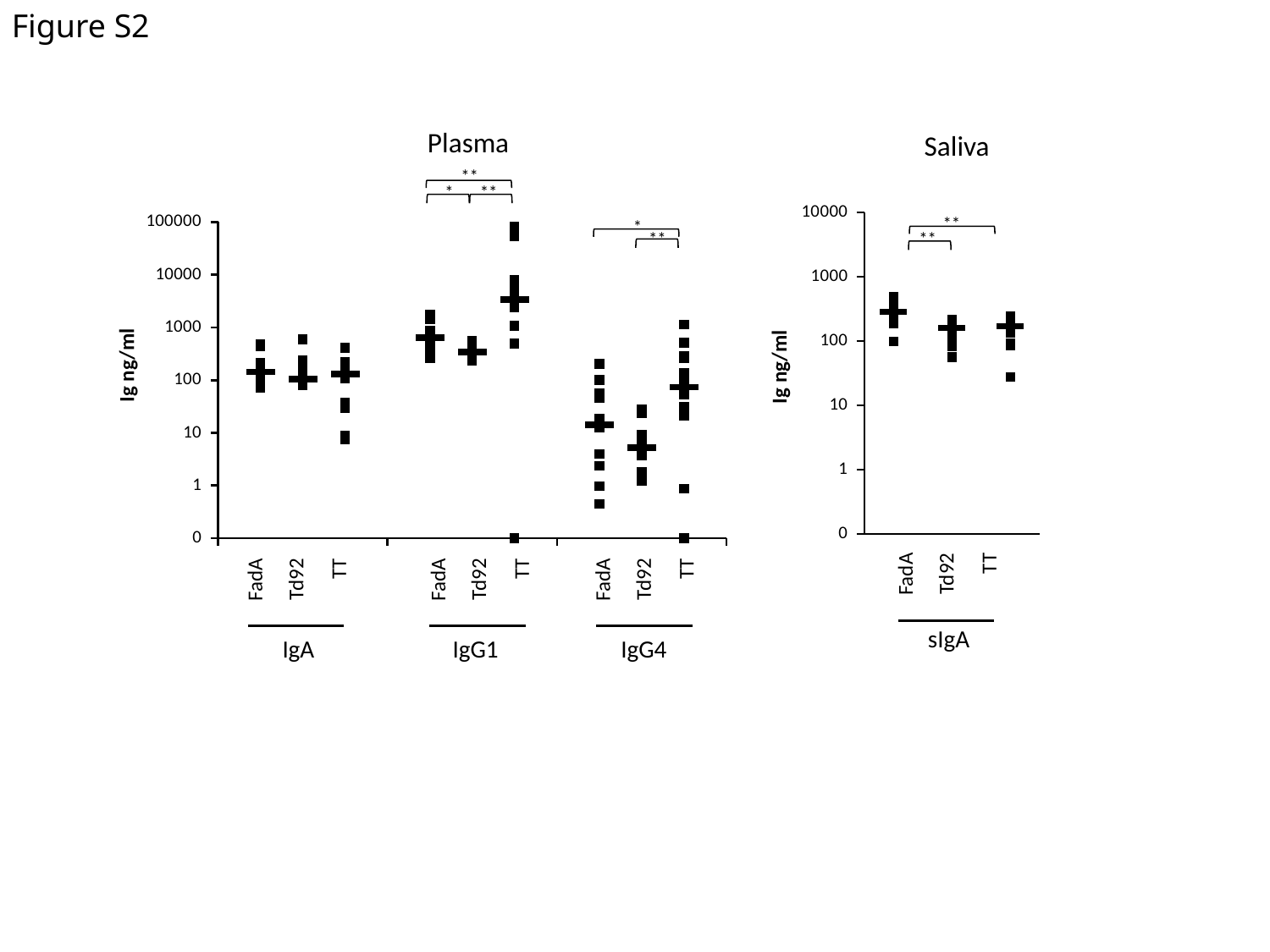

Figure S2
Plasma
Saliva
**
**
*
### Chart
| Category | | | | | | | | | | | | | |
|---|---|---|---|---|---|---|---|---|---|---|---|---|---| **
### Chart
| Category | | | | | | | | | | | | |
|---|---|---|---|---|---|---|---|---|---|---|---|---| *
 **
**
Ig ng/ml
Ig ng/ml
TT
TT
TT
FadA
Td92
FadA
Td92
FadA
Td92
IgA
IgG1
IgG4
TT
FadA
Td92
sIgA
