# Supplementary material for: Antibody and T Cell Responses to Fusobacterium nucleatum and Treponema denticola in Health and Chronic Periodontitis
Source: PLoS One. 2013 Jan 15;8(1):e53703. doi: 10.1371/journal.pone.0053703 (PMC3546045; doi:10.1371/journal.pone.0053703)
Supplement: Figure S3 — Comparison of cytokine response of PBMCs to FadA, Td92, and TT in healthy individuals. From the results shown in Figure 3, the data of healthy subjects are graphed separately. *, P<0.05; **, P<0.01 compared to No Ag. #, P<0.05; ##, P<0.01. (PPTX) [file pone.0053703.s003.pptx]

## Slide 1
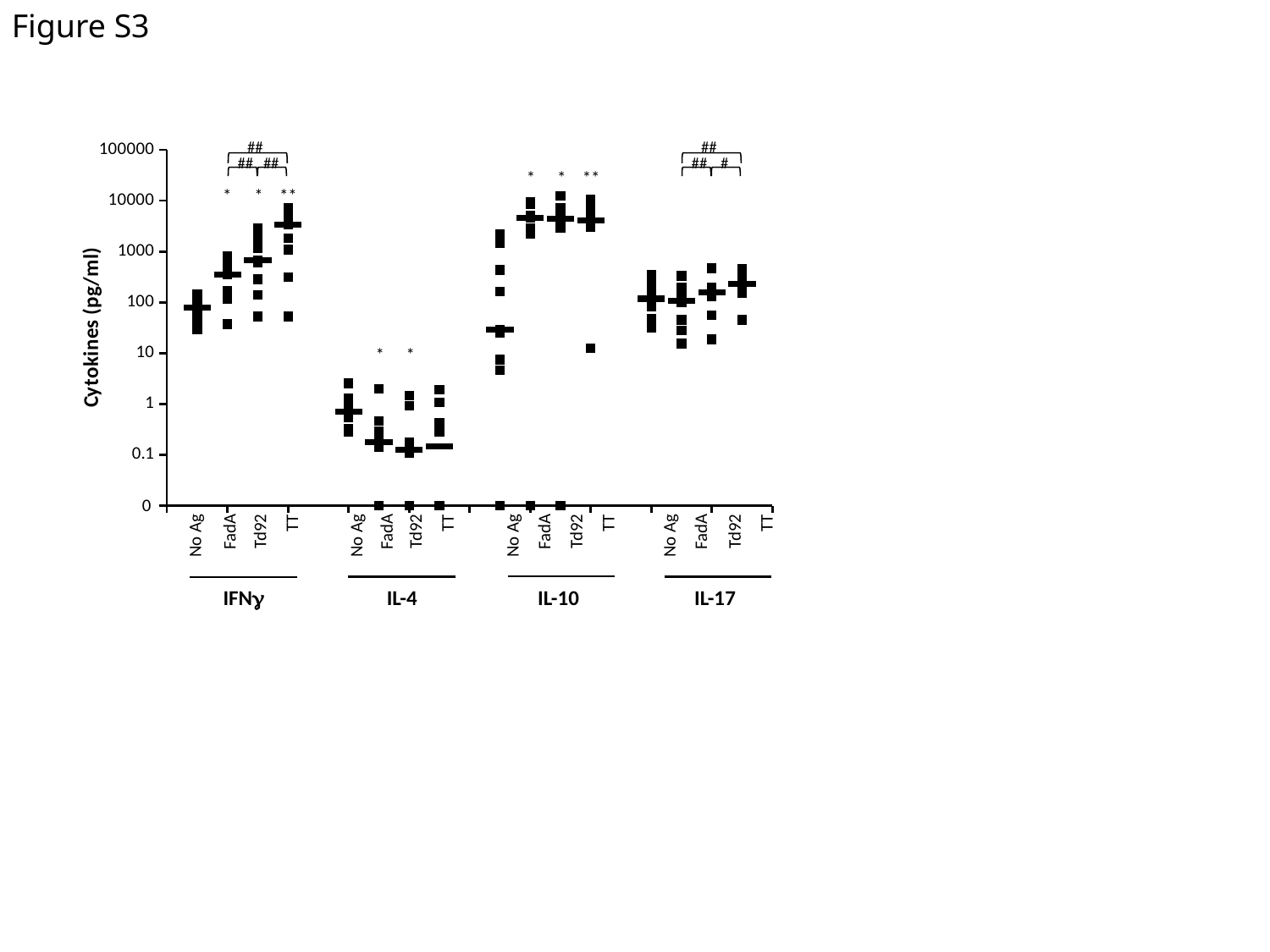

Figure S3
##
##
 ##
##
#
 ##
### Chart
| Category | | | | | | | | | | | | |
|---|---|---|---|---|---|---|---|---|---|---|---|---|*
*
**
*
*
**
*
*
0
TT
TT
TT
TT
FadA
Td92
FadA
Td92
FadA
Td92
FadA
Td92
No Ag
No Ag
No Ag
No Ag
IFNg
IL-4
IL-10
IL-17
